# Prenatal diagnosis of non-typical Chiari malformation type I associated with de novo Nuclear Factor I A gene mutation: a case report

**DOI:** 10.1186/s13256-024-04361-1

**Published:** 2024-02-13

**Authors:** Xuan-Hong Tomai, Huu-Trung Nguyen, Thanh-Truc Nguyen Thi, Tuan-Anh Nguyen, Thuy-Vy Nguyen

**Affiliations:** 1 University of Alberta, Alberta, Canada; 2 University of Nam Can Tho, Can Tho, Vietnam; 3 University of Medicine and Pharmacy, Ho Chi Minh City, Vietnam; 4 University Medical Center, Branch 2, Ho Chi Minh City, Vietnam; 5 University of Sciences, Ho Chi Minh City, Vietnam

**Keywords:** Case report, Chiari malformation type I, NFIA gene mutation, Pregnancy, Prenatal diagnosis

## Abstract

**Background:**

Chiari malformation is one of the most common Central nervous system (CNS) abnormalities that can be detected in routine fetal scanning. Chiari malformation type I (CMI) is a congenital defect characterized by a displacement of the cerebellar tonsils through the foramen magnum. The etiology of CMI has not been well established and suggested having multifactorial contributions, especially genetic deletion. Clinical characteristics of this anomaly may express in different symptoms from neurological dysfunction and/or skeletal abnormalities in the later age, but it is rarely reported in pregnancy.

**Case presentation:**

We present a case in which the Chiari malformation type I was diagnosed with comorbidities of facial anomalies (flatting forehead and micrognathia) and muscular-skeletal dysmorphologies (clenched hands and clubfeet) at the 24^+6^ weeks of gestation in a 29-year-old Vietnamese pregnant woman. The couple refused an amniocentesis, and the pregnancy was followed up every 4 weeks until a spontaneous delivery occurred at 38 weeks. The newborn had a severe asphyxia and seizures at birth required to have an emergency resuscitation at delivery. He is currently being treated in the intensive neonatal care unit. He carries the novel heterozygous *NFIA* gene mutation confirmed after birth. No further postnatal malformation detected.

**Conclusion:**

CMI may only represent with facial abnormalities and muscle-skeletal malformations at the early stage of pregnancy, which may also alert an adverse outcome. A novel heterozygous NFIA gene mutation identified after birth helps to confirm prenatal diagnosis of CMI and to provide an appropriate consultation.

## Background

Chiari malformation (CM), one of the congenital disorder in Central nervous system (CNS) anomalies, is characterized by downward displacement of the brain and hindbrain including cerebellum, pons, and medulla oblongata in the underdeveloped posterior fossa, and it can be observed in routine prenatal scanning during pregnancy [1–3]. The CM has been classified into four types according to the severity of the brain component that protrude into the spinal canal [4]. The CM type I (CMI) involves the cerebellar tonsils pushing into the foramen magnum. This condition may be diagnosed in the context of neurological dysfunction caused by neural tissues compression leading to syringomyelia, hydrocephalus, scoliosis, as well as craniovertebral instability [4–7]. Whereas the CM type II, also called Arnold-Chiari II malformation, and CM type III present severe neurological issues because both cerebellum and brain tissue prolapsing into the foramen magnum (CM type II) or herniating through an abnormal opening in the back of skull (CM type III) [2, 4]. CM type IV is very rare, characterized by cerebellum is not fully developed but is in a normal position in the posterior fossa [3].

Up to 70% of CMI, also called non-syndromic CMI, have no special findings in prenatal ultrasound [1, 8]. Neural tissue compression, the most common symptom of CMI, is caused by either congenital reduced volume of posterior fossa or acquired condition resulted from a syringomyelia development within the cerebrospinal fluid [4, 7]. That is why this disorder has no significant symptom at the early stage and becomes symptomatic when there is a direct compression of the neural tissue at the craniovertebral junction or an obstruction of cerebrospinal fluid flow dynamics [6]. Consequently, CMI may have diverse clinical findings depended on the level of neural tissue compression above or below of the craniovertebral junction [8, 9]. In the literature, CMI has been well documented in addition to comorbidities such as the neurological dysfunction in syringomyelia (in 25% of cases) [5], scoliosis (in 20%) [10], hydrocephalus (in 10%) [11], multiple congenital anomalies (4%) [8] or even neuropsychiatric disorders in children or adults [8]. When CMI is associated with multiple congenital anomalies, known as syndromic CMI, this disorder may represent an early-onset condition, which can predict an adverse outcome, especially in case of CMI with syringomyelia [1, 6].

The etiology of CMI remains unclear but it could be attributed to a combination of multi-genetic mutations [6, 8]. For example, a specific *FGFR2* gene mutation is documented in CMI with syringomyelia [12], and a *PAX2* gene mutation is reported in CMI with renal-coloboma [13]. Frequently, a novel *NFIA* nonsense gene mutation is also shown a relation to CMI with or without brain abnormalities and urinary tract defects [14–17]. Understanding the underlying etiologies of CMI and its comorbidities helps to better predict adverse outcomes [8], as well as provide a detailed management approach in prenatal and postnatal consultation.

To our knowledge, there are only two cases of CMI reported in pregnancy, which demonstrated very poor prognosis. In the first case described by Iruretagoyena et al., where a fetus was diagnosed with CMI accidentally when detected abnormalities of fetal limbs’ position (spastic posture) in the scanning at 18 weeks of gestation. This fetus died at 4 weeks later because of enlarged syringomyelia within the cerebrospinal space. No gene mutation was mentioned in this case [1]. The second case reported by Chen et al., in which ultrasound findings at 30 weeks of pregnancy showed brain malformation with macrocephaly, ventriculomegaly, corpus callosum hypogenesis, micrognathia and ambiguous external genitalia. A chromosome 1p32-p31 deletion syndrome was confirmed and fetus was dead after 3 weeks later [17].

Here we present the diagnosis of CMI associated with multi-congenital abnormalities at 24^+6^ weeks of gestation. The fetus represented a mild ventricular dilatation, facial dysmorphologies (flattening forehead, anteverted nostrils, and micrognathia) and limbs abnormalities (clenched hands and clubfeet). Amniocentesis for fetal karyotype was refused, but the newborn was identified to be carrying a *NFIA* gene mutation after birth. Baby underwent an emergency resuscitation because of asphyxia and seizures at birth and he has been treated in the neonatal intensive care unit (NCIU). He has not shown any signs of improvement. Based on the case report, we review the literature, highlight the effective approach to diagnose an early-onset CMI, and enumerate clinical-based evidence in predicting fetal outcomes.

## Case presentation

A 29 year-old Vietnamese pregnant woman, gravida 0 parity 0, was transferred to our hospital to perform a routine fetal anatomy scanning at 24^+6^ weeks of gestation. There was no remarkable history of chronic diseases and congenital abnormalities in the couple’s family. Both pregnant woman and her partner are healthy and identified as Southeast Asian and belonged to the “Kinh” ethnicity in Vietnam.

The routine first trimester screening with non-invasive Prenatal test (NIPT) results showed no chromosomal aberration for trisomy 13, 18 and 21.

**Fig. 1:**
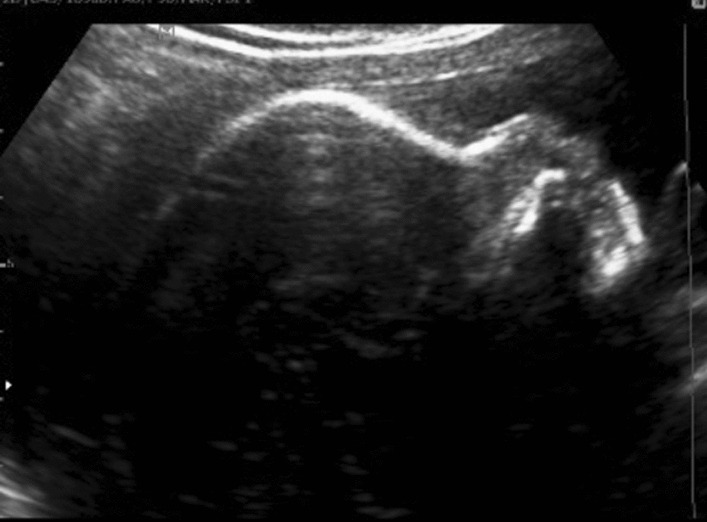
Flattening forehead and micrognathia

**Fig. 2:**
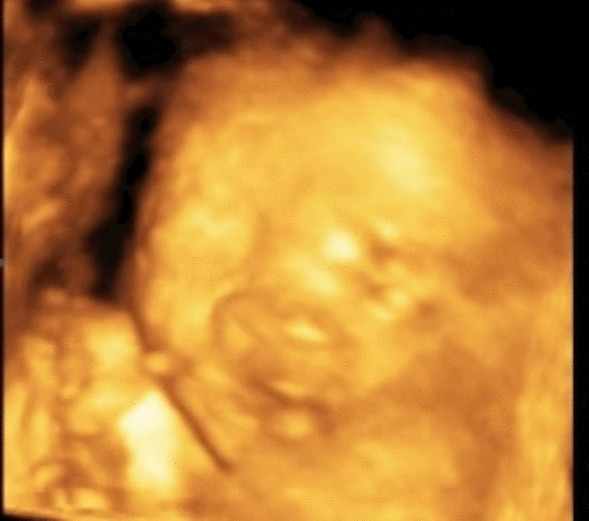
Small and anteverted nostrils

**Fig. 3:**
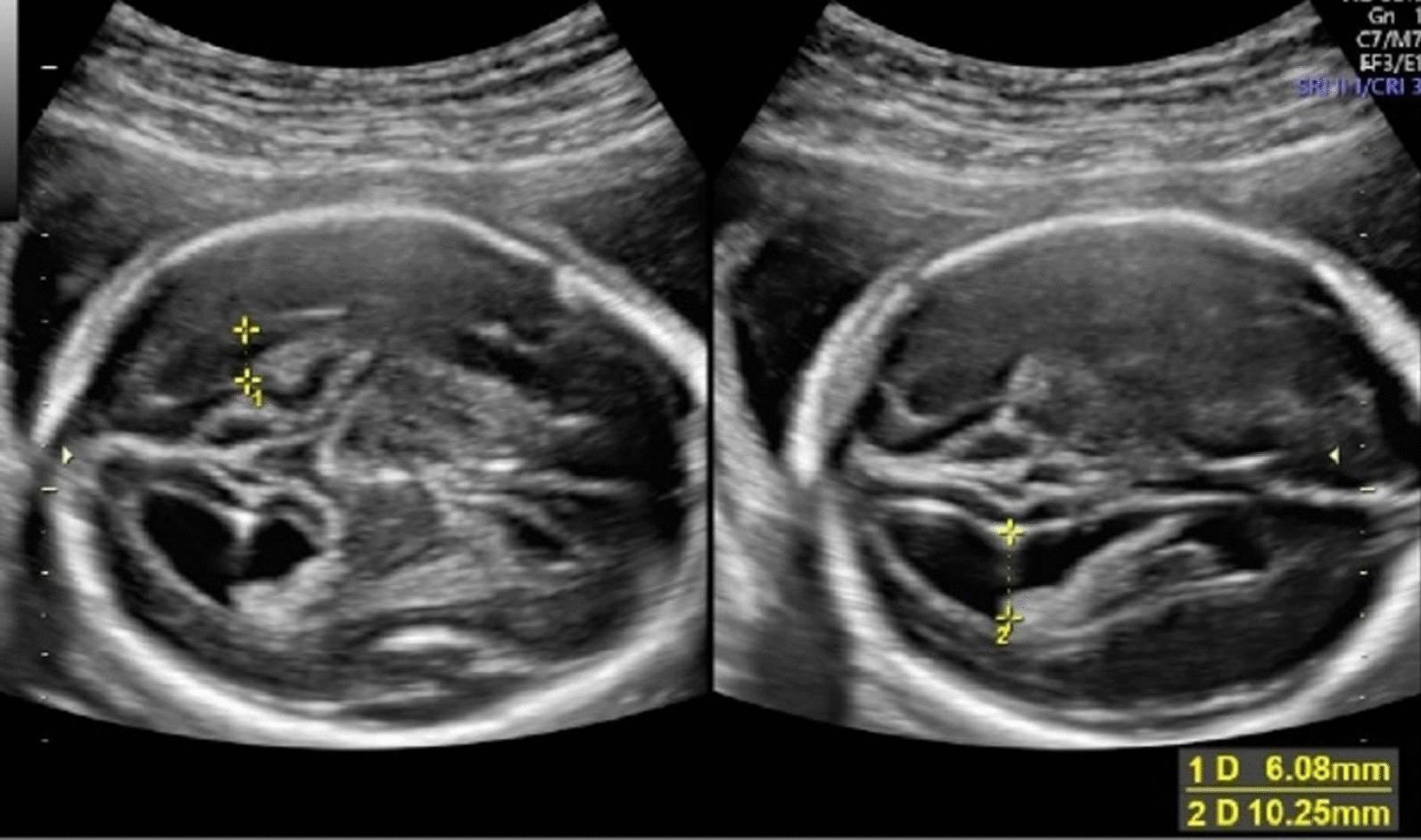
Mild unilateral ventricular dilatation

**Fig. 4:**
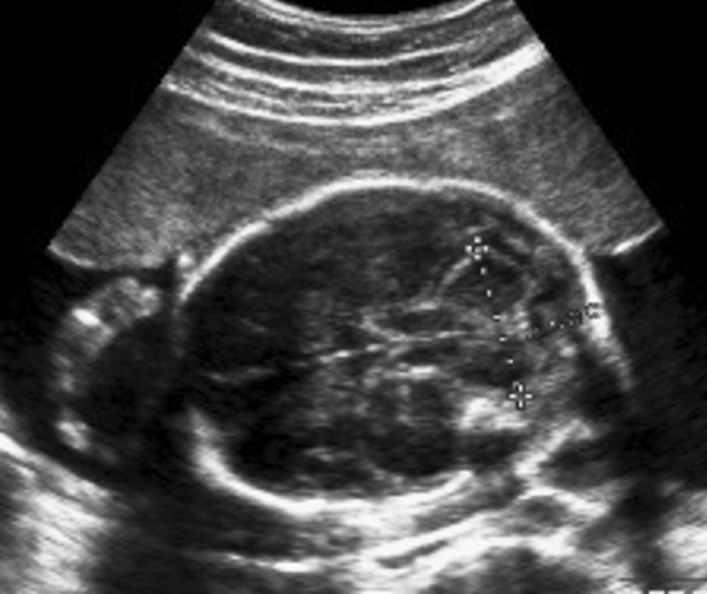
Normal cistern magna and nuchal fold

Prenatal ultrasound at 24^+6^ weeks of gestation revealed flatting of the forehead and micrognathia (Fig. 1), small and anteverted nostrils (Fig. 2*),* mild dilated ventriculomegaly (10.25 mm) with a normal corpus callosum (Fig. 3)*,* normal cistern magna and nuchal fold (Fig. 4), clubfeet and clenched hands. A diagnosis of Chiari malformation was suspicious, and the couple underwent a genetic counseling, but they declined an amniocentesis to evaluate the fetal karyotype due to religious faith.

**Fig. 5:**
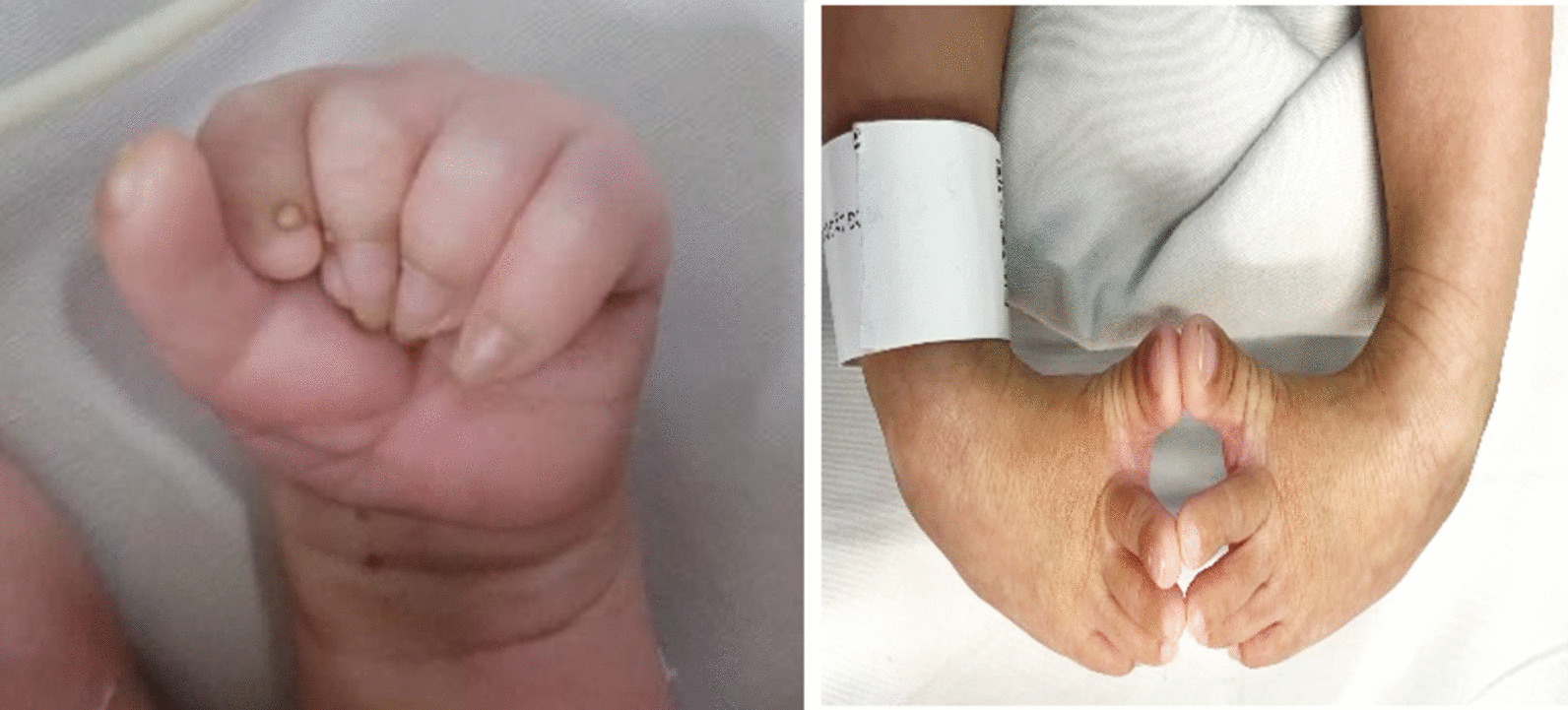
Newborn with clenched hands and clubfeet

The pregnancy continued to be monitored in the private antenatal clinic. Fetal anatomy and development were checked every 4 weeks, and there was no further abnormal findings detected. Baby was born at 38 weeks of gestation after 6 h of spontaneous labor through the vaginal delivery. The boy had normal growth with a birth weight of 3200 g, birth length of 50 cm, head circumference 33 cm, and thorax circumference 32 cm. He was presented in cyanic asphyxia status and seizure at birth. APGAR’s score was 3 at 1st minute, then increased 7 at the 3rd minute and 8 at 5th minutes after an emergency resuscitation. Baby’s facial malformation and limbs deformities (clenched hands and clubfeet) were consistent with prenatal diagnosis (Fig. 5).

The neonatalist recognized no additional anomaly at birth. A heart and abdominal ultrasound showed in normal range. MRI was performed on the 10th day afterbirth and detected mild ventricular dilatation (14 mm), micrognathia, mild dilatation of cortical fissure without brain injury. There was no sign of protruding cerebellar tonsils into the foramen magnum.

A targeted next-generation sequencing (xGen Inherited Diseases Hyb Panel, IDT, USA) and bioinformatics with QIAam DNA Blood Mini Kit (Qiagen) and Genome Analysis Toolkit were used to identify the newborn’s gene mutation. After applying an expanded carrier screening with a panel of 540 genes, the newborn’s karyotype was determined 46, XY and the *NFIA* gene mutation was also detected. Compound heterozygous *NFIA* genes were found in chromosome 1 with two variants NM_001134673.4: c.1255-2A > C (p.?) and NM_001134673.4: c.1255-1G > C (p.?). These variants are pathogenic type. Newborn’s parents were invited to have the target next-generation sequencing for NFIA and both results were negative.

Baby’s breathing has been assisted with the ventilator machine in the NICU and he still is in a critical condition.

## Discussion and conclusions

Chiari malformations are structural defects including abnormal posterior fossa and hindbrain (cerebellum, pons, and medulla oblongata) [4]. While CM type II and type III are the early onset congenital malformations with typical ultrasound findings of obliteration of posterior fossa, banana-shaped cerebellum, and meningoencephalocele [2, 4], CMI is the least severe disorder and generally found asymptomatic and rarely diagnosed during pregnancy [4]. In term of clinical practice, CMI has been divided into two types: non-syndromic CMI, also known as an idiopathic disorder and syndromic CMI, which is associated with comorbidities or known genetic syndromes [8]. The non-syndromic CMI has occupied 71% in CMI and often found incidentally in later age in both children and adults through the symptoms of scoliosis and common neuropsychiatric disorders such as developmental delay or autism [8]. In contrast, syndromic CMI is the earliest onset CMI that may be detected with neurological dysfunction (17%), or skeletal abnormalities (8%), or multiple congenital anomalies (4%) including urinary defects [8].

In the prenatal diagnosis of syndromic CMI, despite it is an early onset disorder, it is still challenging to diagnose. The reason is that the ultrasound findings are rarely documented [1] and this disorder may represent in the context of infinite diseases [6, 8, 9]. To date, there are only two cases illustrated a syndromic CMI detected during pregnancy. Both cases have affirmed that an early onset syndromic CMI may predict an adverse outcome. In the present case, the fetus demonstrated different pathological characteristics including a mild brain malformation (mild ventriculomegaly), prominent facial abnormalities (a flat forehead, micrognathia, and small and anteverted nostrils) associated with limb deformities (clenched hands and clubfeet) at 24^+6^ weeks of gestation. The ultrasound findings of facial and musculoskeletal anomalies in our case were not giving strong evidence to confirm a typical syndromic CMI, where callosum hypogenesis and/or urinary tract defect were often present. However, in facing with the newborn with combined multi-congenital anomalies with dominant malformations in face and limbs and cyanic asphyxia, we are thinking about the existence of nervous system disorder caused by a tissue compression above the craniovertebral junction. A severe birth asphyxia and seizures required an emergency resuscitation could be explained by an underlying hindbrain and cerebellum compression may cause the hypoactivity of the interspatial and respiratory muscles.

So far the etiology of CMI has not been well-established [8], understanding the underlying etiology of CMI is another challenge, but it helps to improve the quality of prenatal diagnosis and provide a better management approach. Up to 50 disorders associated with CMI have made more difficult to identify the prevalence and etiology of this congenital malformation [8, 9]. Luckily, the role of molecular cytogenetic evaluation has been affirmed as a valuable tool to establish a diagnosis of an early onset syndromic CMI [17]. Many gene mutations in the chromosome 1 and 22 including *FGFR2, PAX2, NFIA* have been identified underlying causes of CMI [13–18]. Among those gene mutations, *NFIA* gene (OMIM 600727) locates at 1p31.3-p31.2 and encodes Nuclear Factor IA (*NFIA)* protein [17] has showed an important role in the development of the CNS through the axon guidance with outgrowth, glial or neuronal cell differentiation, and neuronal migration [19, 20]. That is why a *NFIA*-related disorder is often characterized by CNS abnormalities including brain and facial malformations, dysmorphologies of long and flat bones, and genito-urinary tract defects [15, 21]. Those clinical findings are consistent with both non-syndromic and syndromic CMI [7, 18].

In recent case report, postnatal genetic test identified compound heterozygous *NFIA* gene mutation in pathogenic type [variants named c.1255-2A > C (p.?) and c.1255-1G > C (p.?)]. Based on both clinical signs, imaging findings and genetic test, we can confirm that the newborn was suffering from syndromic CMI with a presence of *NFIA* gene mutation [22].

*NFIA*-related disorder is inherited in an autosomal dominant condition, so a baby of either parent affected by *NFIA* gene mutation has a 50% chance of suffering this disorder via inheritance of a dominant allele. In our case, a compound heterozygous *NFIA* gene mutation detected in newborn’s blood test can be considered as a novel genetic mutation because both parents are healthy and do not have the history of *NFIA-*related disorder noticed in their family. Moreover, they were performed the target next-generation sequencing, then their result showed negative for *NFIA*. Our finding is consistent with what Senaratne et al. reported, who also stated that the proportion of de novo* NFIA* gene mutation is around 75–80% and it depends on the causative gene alteration [23].

It is obvious that the changing of diverse pathologic genes has assigned different characteristics of dysmorphic features during pregnancy and growth development. Recently, Bertini et al. has affirmed the key role of *NFIA* gene in various differentiation pathways during embryogenesis and suggested a careful fetal dysmorphic examination, especially in craniofacial features and hand malformations, when there is a *NFIA* gene mutation determined [24]. Similarly, Bayat et al. reported four cases identified *NFIA* gene mutation with typical syndromic CMI combined with brain malformation and urinary tract defect [25]. However, Iossifov et al. described an infancy carried a novel truncating, heterozygous *NFIA* mutation with characteristics of autism spectrum (neuropsychiatric) disorder without brain malformation or urinary tract defects [26]. Our case is the first report of syndromic CMI diagnosed at the second trimester of pregnancy. This congenital disorder is also caused by a novel compound heterozygous *NFIA* gene mutation, but it has a different phenotypic trait: syndromic CMI without brain malformation. Our findings have added the new evidence of syndromic CMI with de novo* NFIA* gene mutation in the literature of prenatal diagnosis.

A prenatal diagnosis of syndromic CMI may only represent with facial abnormalities and muscle-skeletal malformations. An early onset CMI may alert adverse outcomes in pregnancy. It is recommended to combine prenatal ultrasound and molecular genetic analysis to confirm syndromic CMI, understand its etiology, and provide an appropriate consultation and treatment approach to improve the quality of perinatal and postnatal care.

## Data Availability

The dataset of the current study is available from the corresponding author upon motivated request.

## Abbreviations

CNS: Central nervous system
CM: Chiari malformation
CMI: CM type I
MRI: Magnetic resonance imaging
NCIU: Neonatal intensive care unit
